# Supplementary material for: Long COVID-19 in children: an Italian cohort study
Source: Ital J Pediatr. 2022 Jun 3;48:83. doi: 10.1186/s13052-022-01282-x (PMC9163526; doi:10.1186/s13052-022-01282-x)
Supplement: Supplementary file 1 — Additional file 1: Supplementary Table 1. Cumulative incidence of Long COVID-19 symptoms and signs, as a function of age, in hospitalized children. Supplementary Figure 1a-1b. Cumulative incidence of Long COVID-19 symptoms in the hospitalized patients, as a function of previous diseases. Supplementary Table 2. Independent effect of setting, gender, age, and symptoms during SARS-CoV-2 infection on the Long COVID-19 symptoms. [file 13052_2022_1282_MOESM1_ESM.docx]

**LONG COVID-19 IN CHILDREN: AN ITALIAN COHORT STUDY**

Gianfranco Trapani [1], Giuseppe Verlato [2], Enrico Bertino [3], Giulia Maiocco [3], Roberta Vesentini [2], Alessia Spadavecchia [3], Angelica Dessì [4] and Vassilios Fanos [4]

**Corresponding author**: Giulia Maiocco: giulia.maiocco@unito.it

**For the of Primary Care Pediatrician Research Group:** Al Jamal O, Casti T, Zanino L, Sottemano S, Careddu D, Bembo V, Franchi G, Muià CC, Macrì F Tiozzo V, Camilli S, Meleleo D, Lombardi Mistura L, Romeo L, Brazzoduro V, Soldato G, Lanza C, Piasenti C, Ragnatela G, Minchilli G.

**AFFILIATIONS**

**[1]** Alfred Nobel International Association Studies Center, Sanremo, Italy.

**[2]** Unit of Epidemiology and Medical Statistics, Department of Diagnostics and Public Health, University of Verona, Verona, Italy.

**[3]** Department of Public Health and Pediatric Sciences of the University, City of Health and Science of Turin, Turin, Italy.

**[4]** Department of Surgical Sciences, University of Cagliari and Neonatal Intensive Care Unit, AOU Cagliari, Cagliari, Italy

**ABSTRACT**

**BACKGROUND:** Long COVID-19 syndrome is a complex of symptoms that occurs after the acute SARS-CoV-2 infection, in the absence of other possible diagnoses. Studies on Long COVID-19 in pediatric population are scanty and heterogeneous in design, inclusion criteria, outcomes, and follow-up time. The objective of the present study is to assess the prevalence of Long COVID-19 syndrome in a cohort of Italian pediatric primary care patients, observed for a period of time of 8 to 36 weeks from healing. Prevalence was also assessed in a cohort of pediatric patients hospitalized during acute infection.

**METHODS:** Data concerning 629 primary care patients with previous acute SARS-CoV-2 infection were collected by a questionnaire filled in by Primary Care Pediatrician (PCP). The questionnaire was administrated to patients by 18 PCPs based in 8 different Italian regions from June to August 2021. Data concerning 60 hospitalized patients were also collected by consultation of clinical documents.

**RESULTS:** Cumulative incidence of Long COVID-19 resulted to be 24.3% in primary care patients and 58% in hospitalized patients. The most frequently reported symptoms were abnormal fatigue (7%), neurological (6.8%), and respiratory disorders (6%) for the primary care cohort. Hospitalized patients displayed more frequently psychological symptoms (36.7%), cardiac involvement (23.3%), and respiratory disorders (18.3%). No difference was observed in cumulative incidence in males and females in both cohorts. Previous diseases did not influence the probability to develop Long COVID-19. The prevalence of Long COVID-19 was 46.5% in children who were symptomatic during acute infection and 11.5% in asymptomatic ones. Children aged 0 to 5 years had a greater risk to develop respiratory symptoms, while adolescents (aged 11-16 years) had a greater risk to develop neurological and psychological Long COVID-19 symptoms.

**CONCLUSIONS:** Our study demonstrates that Long COVID-19 is a reality in pediatric age and could involve even patients with mild or no acute symptoms. The results stress the importance of monitoring primary care pediatric patients after acute COVID-19 infection and the relevance of vaccination programs in pediatric population, also in order to avoid the consequences of Long COVID-19 syndrome.

**Keywords: Long COVID-19; primary care assisted children; COVID-19 in pediatric age**

**BACKGROUND**

Despite more than 10,000 articles are available nowadays on Pub Med on the Long COVID-19 issue, data and interpretations about this clinical condition are still not clear, especially in pediatric age.

The World Health Organization estimates that approximately 25% of adult patients with COVID-19 infection may develop persistent symptoms [1]. An Italian study shows that persistence of at least 2 symptoms after acute infection is evident in 32% of patients, and of at least 3 symptoms in 55% [2]; a Chinese study, on the other hand, observes the presence of Long COVID-19 in 76% of patients after 6 months from the detection of the infection [3]. However, a precise assessment of the extent of this phenomenon is difficult, due to the differences in methods, definitions, and population detectable between the different studies.

The Long COVID-19 syndrome defines specifically a condition characterized by the presence of symptoms related to SARS-CoV-2 infection which appear or persist after the acute event, in the absence of alternative diagnoses [1].

In adults, Long COVID-19 is represented by a heterogeneous set of clinical symptoms derived from the involvement of multiple systems: respiratory, cardiovascular, nervous, gastrointestinal, renal, hematological, endocrine, and cutaneous ones [1]. The most frequently observed manifestations include severe and persistent fatigue, cough, anorexia, and fever; neurological (anosmia and ageusia) or neuropsychiatric manifestations are possible [4,5].

The Long COVID-19 syndrome has also been described in children, though less frequently than in adults. In this context, it is important to distinguish Long COVID-19 from Multisystem Inflammatory Syndrome in Children (MIS-C), which represents an acute condition characterized by multisystem organ involvement ­– together with fever and laboratory evidence of inflammation – arising in a child currently or recently tested positive for SARS-CoV-2 infection [6]. It is not possible to make a reliable epidemiological estimate of Long COVID-19 syndrome in the pediatric population, as the data currently available are based on heterogeneous studies performed almost exclusively in the US and the UK, on hospitalized subjects. European data are still insufficient, particularly with reference to the primary care setting.

The aim of the present study is to estimate the prevalence of Long COVID-19 syndrome in pediatric age: principally in a cohort of subjects with access to primary care and with an observation time from 8 to 36 months after recovery; secondly in a cohort of pediatric subjects with hospital access.

**METHODS**

**Population**

In Italy the National Healthcare System provides that every child (from 0 to 16 years of age) refers to the Primary Care Pediatrician (PCP) for health issues, thus pediatric clinics on territory represent the first level of care in pediatric age.

Our study involved 18 pediatric clinics in 8 Italian Regions (Liguria, Piedmont, Lombardy, Emilia-Romagna, Lazio, Puglia, Calabria and Sardinia) which globally take care of a pediatric population of 21,663 children. Patients on territory were compared with a series of patients (0 to 16 years of age) admitted to the Vittore Buzzi Children's Hospital in Milan, Lombardy.

The data collected belong to patients who acquired SARS-CoV-2 infection in the period October 2020-June 2021, and the observation time ranges from 2 to 9 months after recovery from acute infection.

Inclusion criteria were being diagnosed with COVID-19 by a positive molecular swab and being healed with negative molecular swab from at least 8 weeks.

**Questionnaire**

The survey was conducted in the quarter June-August 2021, and a questionnaire (**Appendix**) was proposed for every recruited case. The questionnaire consists of questions with mandatory response: the first part is addressed directly to PCPs, and aim to identify their Region of origin, the total number of their patients and the number of patients who tested positive for SARS-CoV-2 in the considered period. In the second part, pediatricians collected information from the parents of every enrolled patient regarding: age and sex of the child, time elapsed from recovery from COVID-19, possible previous chronic diseases, and health conditions following clinical recovery from COVID-19. Specifically, the frequency of respiratory diseases, gastrointestinal disorders, social anxiety, depression episodes, learning disabilities, eating disorders, headache, insomnia, tachycardia, muscular and joint pain, abnormal fatigue, cutaneous manifestations, hair loss, ageusia, and anosmia, and potential presence of any other symptoms, are investigated. The PCPs completed the online questionnaire during telephone consultations, or directly in pediatric primary care clinic. Data relating to hospitalized patients were extrapolated by hospital pediatricians from medical records.

Long COVID-19 syndrome was assumed when at least one of the above reported manifestations increased in frequency during the 8-36 weeks after recovery from SARS-CoV-2 infection, with respect to the previous year.

Written informed consent was obtained from all parents and/or legal guardian of enrolled children. All questionnaires were anonymous. Collected data were entered on a Limesurvey platform with an encrypted and protected database made freely available by the University of Verona. The study was approved by the Regional Ethics Committee of Liguria on the 16^th^ of June 2021 with protocol number 419/2021 - id 11611. All the methods were carried out in accordance with the ethical standards as laid down in the 1964 Declaration of Helsinki and its later amendments.

**Statistical Methods**

The distribution of age by center was described by box-and-whisker plots. Fisher exact test was used to evaluated significance of differences in Long COVID-19 symptoms between primary care children and hospital series, between genders and among age classes (0-5, 6-10, 11-16 years).

A weighted prevalence of Long COVID-19 in Italy was computed, using as weights the size of pediatric population (0-16 years) in Northern/Central/Southern Italy, amounting to 4,031,023 / 1,698,481 / 3,051,936 respectively (demo.istat.it).

Determinants of Long COVID-19 symptoms were further investigated by a multivariable logistic model, where symptoms were the response variable, gender, age class, setting (primary care versus hospital), pre-existing diseases, COVID-19 symptoms (yes/no) the explanatory variables. To ensure enough power to statistical analysis, only those symptoms exceeding 40 cases (at least one symptom, respiratory / psychological / neurological symptoms, fatigue) were considered as response variables.

Analyses were performed with STATA statistical software, release 17 (StataCorp, College Station, TX, USA) and statistical significance was set at p < 0.05.

**RESULTS**

**Description of the study sample**

1351 cases of positive children were reported, equal to 6% of the pediatric population in the 8 Regions participating in the survey. 1289 were the primary care cases, and 62 were the hospitalized ones. Fulfilled questionnaires were 689, of which 629/1289 (49%) relating to children in primary care and 60/62 (97%) to hospitalized children. There was no difference between males (n = 371; 51.9%) and females (n = 344; 48.1%) participating in the survey.

**Table 1** describes the number of patients (21,663) assisted by the family pediatricians participating in the study, the number of positive cases (1,289), and their prevalence (6%). The number of positive cases participating in the survey (629), the percentage of those who responded to the questionnaire (49%), and the percentage of symptomatic subjects during the acute phase (36.6%) are also reported. Comparing the three major Italian macro-areas (North, Center and South Italy together with Sardinia) we observe that the prevalence of COVID-19 was 5.3% for Northern Italy, 2.4% for Central Italy and 9.2% for Southern Italy and Sardinia. The mean prevalence in Italy was 6%. Of course, this sample, although enrolled in primary care, could be not representative of the whole Italian Pediatric Population. However, the prevalence of COVID-19 was substantially the same, when adjusting for the size of pediatric populations in different Italian macroareas (6.1%).

**Table 1. Description of primary care series.**

|  | **Pediatric population** | **Positive cases** | **COVID-19 prevalence (%)** | **Positive cases participatig in the survey** | **Respondents (%)** | **Symptomatic during acute infection (%)** |
| --- | --- | --- | --- | --- | --- | --- |
| ***Northern Italy*** | ***12303*** | ***653*** | ***5.3*** | ***412*** | ***63.1*** | ***172 (41.7)*** |
| Piedmont | 5303 | 134 | 2.5 | 116 | 86.6 | 54 (46.5) |
| Liguria | 4470 | 399 | 8.9 | 247 | 61.9 | 84 (34) |
| Lombardy | 1100 | 40 | 3.6 | 38 | 95 | 28 (73.7) |
| Emilia-Romagna | 1430 | 80 | 5.6 | 11 | 13.8 | 6 (54.6) |
| ***Central Italy*** | **3320** | **81** | **2.4** | **72** | **88.9** | **32 (44.4)** |
| Lazio | 3320 | 81 | 2.4 | 72 | 88.9 | 32 (44.4) |
| ***Southern Italy and Sardinia*** | **6040** | **555** | **9.2** | **145** | **26.1** | **26 (17.9)** |
| Puglia | 3070 | 90 | 2.9 | 58 | 64.4 | 16 (27.6) |
| Calabria | 1180 | 428 | 36.3 | 53 | 12.4 | 3 (5.7) |
| Sardinia | 1790 | 37 | 2.1 | 34 | 91.9 | 7 (20.6) |
| **Total** | **21663** | **1289** | **6%** | **629** | **49%** | **230 (36.6%)** |

The male to female ratio was close to 1:1 in the primary care setting, as males were 316 (50.2%) and females 313 (49.8%), while hospitalized children had a slightly larger proportion of males (n=34, 56.7%), although the difference was not significant (p=0.348). Hospitalized children were younger than children recruited in primary care: median age was 4 years (p25-p75 = 0-11.5 years) in the former and 8 years (4-11 years) in the latter (p=0.008). Of note, 9/629 children (1.4%) in primary care and 18/60 (30%) in the hospital setting were infant in the 1^st^ year of life. Fifty-nine children (9.4%) in Primary Care and 14 (23%) in the hospital setting had pre-existing disease, and 230 children (36.6%) in Primary Care had presented symptoms during SARS-CoV-2 infection.

Hospitalized children under the first year of life presented high complexity care pathologies.

**Cumulative incidence of Long COVID-19 symptoms**

As shown in **Table 2**, Long COVID-19 symptoms were found in 153 children, recruited in primary care. This figure corresponds to a cumulative incidence of 24.3% Long COVID-19 (95 CI, 21.0-27.9%). Cumulative incidence was more than doubled in hospitalized children (58.3%, 95% CI 44.9-70.9%), with respect to the primary care setting.

In the primary care setting the symptoms most frequently reported as part of Long COVID-19 were abnormal fatigue (7%), neurological (6.8%) and respiratory disorders (6%). In addition, psychological symptoms and muscle/joint pain were reported by 4.9%, loss of taste/smell by 3.3%, and gastrointestinal disorders by 3.0%. Other symptoms were less frequent, being reported by <2% of children with previous SARS-CoV-2 infection.

At variance the most frequent disorders in hospitalized children were psychological symptoms (36.7%), followed by cardiac (23.3%) and respiratory (18.3%) disorders. Other frequently reported disorders were neurological (11.7%), gastrointestinal (10%), and dermatological (10%) disorders.

**Table 2. Cumulative incidence of Long COVID-19 symptoms and signs.**

|  | **Primary care (n=629)** | **Hospital**  **(n=60)** | **p-value*** |
| --- | --- | --- | --- |
| At least one symptom | *153 (24.3)* | *35 (58.3)* | <0.001 |
|  |  |  |  |
| Abnormal fatigue | 44 (7.0) | --- | 0.026 |
| Neurological symptoms | 43 (6.8) | 7(11.7) | 0.188 |
| Respiratory symptoms | 38 (6.0) | 11(18.3) | 0.002 |
| Psychological symptoms | 31 (4.9) | 22 (36.7) | <0.001 |
| Muscle and joint pains | 31 (4.9) | 3 (5.0) | 1.000 |
| Loss of taste/smell | 21 (3.3) | 1(1.7) | 0.712 |
| Gastrointestinal symptoms | 19 (3.0) | 6 (10) | 0.016 |
| Dermatological disorders | 12 (1.9) | 6 (10) | 0.003 |
| Palpitations and cardiac disorders | 5 (0.8) | 14 (23.3) | <0.001 |
| Other symptoms | 4 (0.6) | 19 (31.8) | <0.001 |

*P values were computed by the Fisher’s exact test.

In the present survey parents of children with previous SARS-CoV-2 infection contacted PCPs with increasing frequency as time elapsed since recovery increased: indeed 158 children (25.1%) were seen after 2-3 months, 216 (34.3%) after 4-5 months, and 255 (40.5%) after 6 months and over. The cumulative incidence of Long COVID-19 slightly increased from 20.5% (95% CI 14.3-27.4%) after 2-3 months to 26.9% (21.1-33.3%) after 4-5 months, although the time trend was not significant (p=0.338).

Accordingly, no significant temporal trend was observed as regards specific symptoms of Long COVID-19. Indeed, the cumulative incidence of loss of taste/smell and gastrointestinal/dermatological disorders tended to increase as expected, while the cumulative incidence of respiratory disorders tended to decrease. However, none of these variations achieved statistical significance.

**Gender-age distribution of Long-COVID-19**

The cumulative incidence of Long COVID-19 was about the same in males and females, both in primary care (74/316 = 23.4% and 79/313 = 25.2%, respectively; p=0.642) and in the hospitalized children (20/34 = 59% and 15/26 = 58%; p=1.000).

In the primary care setting the cumulative incidence of Long COVID-19 nearly doubled from 18.3% in children aged 0-5 years to 34.4% in adolescents aged 11-16 years. This trend was confirmed for most Long COVID-19 related disorders (psychological, neurological, loss of taste/smell, cardiac disorders, muscle/joint pain, abnormal fatigue), as shown in **Table 3**. It should be reminded that some symptoms which were rare (abnormal fatigue, neurological symptoms) or absent (loss of taste/smell) in children aged 0-5 years are difficult to reliably assess in this age. At variance, respiratory disorders were inversely related to age, while the cumulative incidence of gastrointestinal and dermatological disorders was not significantly affected by age.

Similar age trends were observed in hospitalized children: the cumulative incidence of Long COVID-19 was 38%, 78% and 84% (16/19) in children aged 0-5, 6-10 and 11-16 years, respectively (p=0.002). An increasing trend with age was observed for most specific symptoms, although statistical significance was achieved only for respiratory, psychological, and cardiac disorders, maybe for the lower sample size (n=60) and statistical power. At variance with primary care setting the cumulative incidence of respiratory disorders increased with increasing age in hospitalized children (**Supplementary Table 1**).

**Table 3. Cumulative incidence of Long COVID-19 symptoms and signs, as a function of age, in the primary care setting.**

|  | **Age class** | | | |
| --- | --- | --- | --- | --- |
|  | **0-5 years**  **(n=202)** | **6-10 years**  **(n=235)** | **11-16 years**  **(n=192)** | **p-value*** |
| At least one Long COVID-19 symptom | 37 (18.3) | 50 (21.3) | 66 (34.3) | **0.001** |
|  |  |  |  |  |
| Abnormal fatigue | 4 (2) | 12 (5.1) | 28 (14.6) | **<0.001** |
| Neurological symptoms | 3 (1.5) | 15 (6.4) | 25 (13) | **<0.001** |
| Respiratory symptoms | 23 (11.4) | 9 (3.8) | 6 (3.1) | **0.001** |
| Psychological symptoms | 5 (2.5) | 7 (3) | 19 (9.9) | **0.001** |
| Muscle and joint pain | 3 (1.5) | 12 (5.1) | 16 (8.3) | **0.005** |
| Loss of taste/smell | 0 | 5 (2.1) | 16 (8.3) | **<0.001** |
| Gastrointestinal disorders | 2 (1) | 8 (3.4) | 9 (4.7) | 0.073 |
| Dermatological disorders | 5 (2.5) | 3 (1.3) | 4 (2.1) | 0.666 |
| Palpitations and cardiac disorders | 0 | 1 (0.4) | 4 (2.1) | **0.043** |
| Other symptoms | 0 | 3 (1.3) | 1 (0.5) | 0.327 |

*P values were computed by the Fisher’s exact test, comparing the three classes of age

Children with pre-existing diseases like various heart disease, atrial and ventricular defect, arterial hypertension, rheumatic carditis, allergic asthma, juvenile idiopathic arthritis, celiac disease, juvenile diabetes, autism, Joubert's syndrome, epilepsy, febrile seizures, epilepsy, imperforate anus, renal malformations (73/689=11%) had a slightly higher cumulative incidence of Long COVID-19 than children without, both in primary care (19/59=32.2% versus 134/570=23.5%; p=0.152) and in the hospital setting (9/14=64% versus 26/46=57%; p=0.760), but the difference was never significant.

Likewise, no significance difference between children with or without pre-existing diseases was detected in Primary Care when considering specific Long COVID-19 related disorders (**Figure 1a-b)**. A borderline significance (p=0.055) was only observed for abnormal fatigue, whose incidence was more than doubled in children with than without pre-existing diseases (8/59=13.6% versus 36/570=6.3%).

**Figure 1a-1b. Cumulative incidence of Long COVID-19 related disorders in the primary care setting (n=629), as a function of previous diseases. Hospitalized patients are described in Supplementary figure 1a-1b.**

**Fig. 1a**


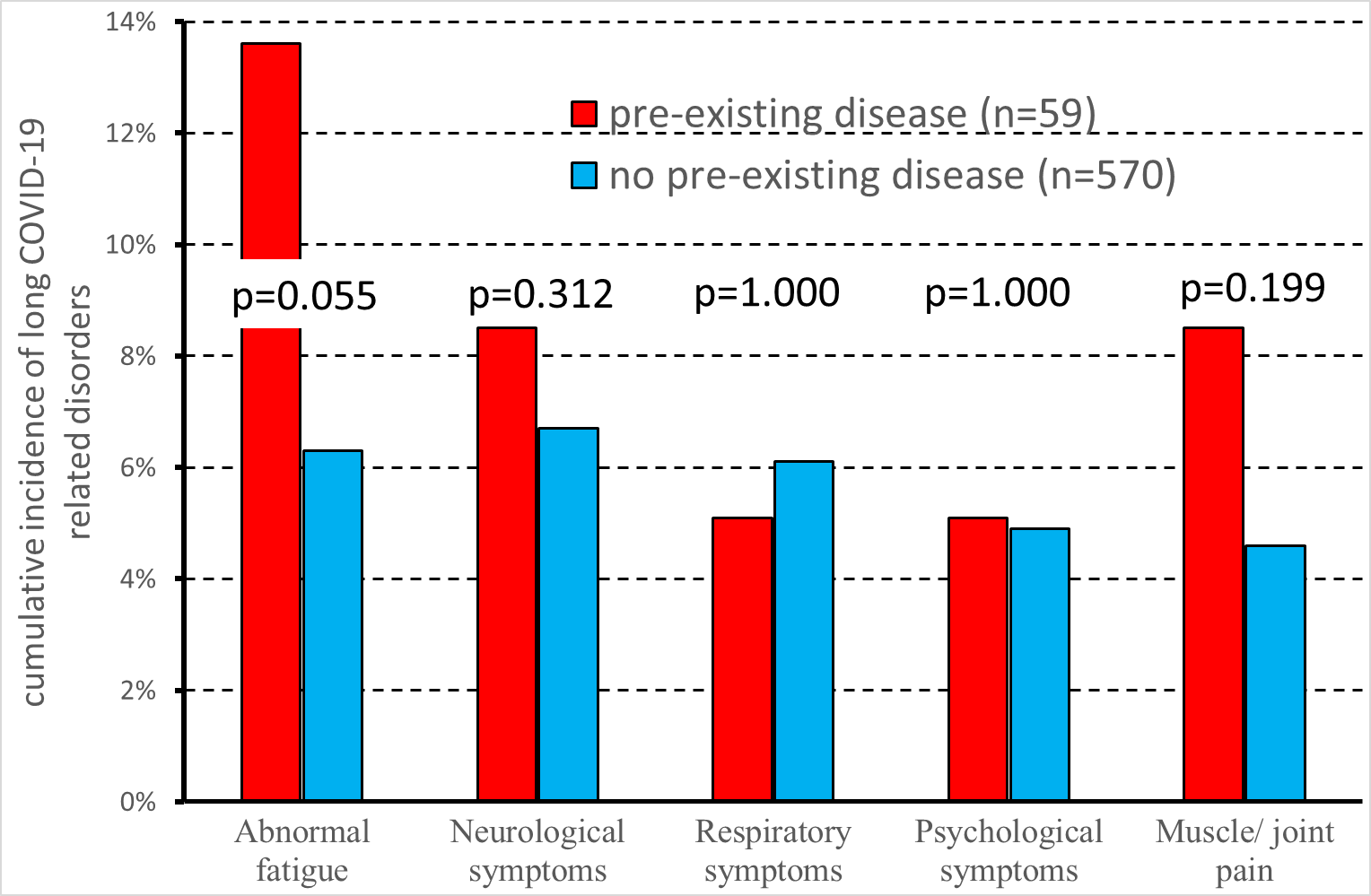


**
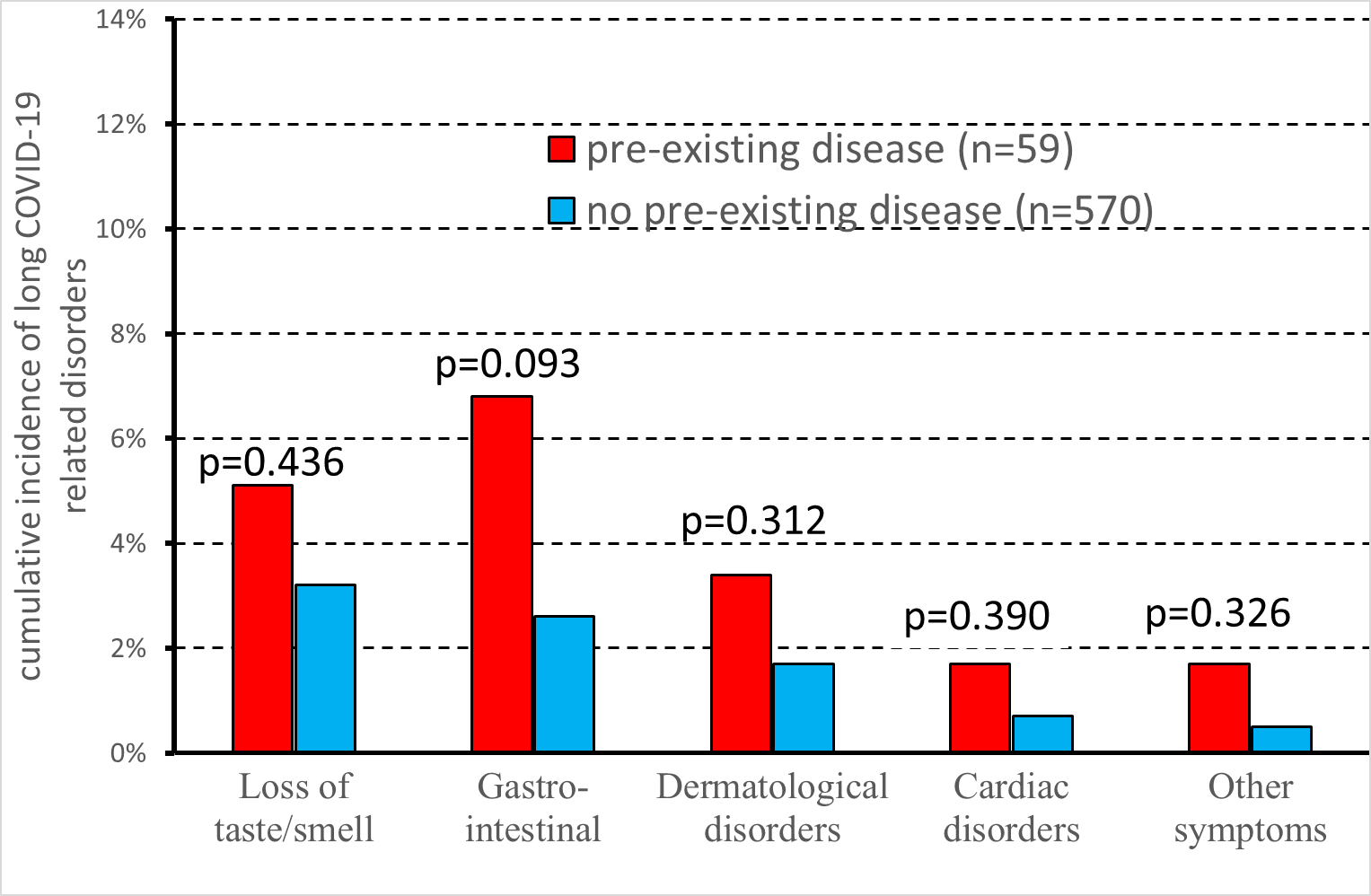
Fig 1b**

**Symptoms during previous SARS-CoV-2 infection**

In primary care Long COVID-19 was strongly associated with previous occurrence of a symptomatic COVID-19 disease, as shown in **Table 4**: cumulative incidence of Long COVID-19 increased from 11.5% (=46/399) in children who had experienced an asymptomatic COVID-19 to 46.5% (=107/230) in those who had had symptoms during the disease (p<0.001).

The association remained significant also when separately considering specific symptoms with at least 10 cases. The difference between symptomatic and asymptomatic children during previous SARS-CoV-2 infection was particularly pronounced when considering abnormal fatigue (16.5% vs 1.5%), neurological disorders (14.4% vs 2.5%), loss of taste/smell (8.7% vs 0.25%), muscle/joint pain (9.6% vs 2.3%). On the other hand, the difference was less marked as regards respiratory (8.7% vs 4.5%) and gastrointestinal (5.2% vs 1.8%) disorders.

**Table 4. Association between symptomatic acute SARS-CoV-2 infection and subsequent development of Long COVID-19 symptoms in the primary care setting.**

| **Long COVID-19 symptoms** | **Symptomatic acute SARS-CoV-2 infection (n=230)** | **Non symptomatic acute SARS-CoV-2 infection (n=399)** | ***p*-value** |
| --- | --- | --- | --- |
| At least one symptom | 107 (46.5) | 46 (11.5) | **<0.001** |
|  |  |  |  |
| Abnormal fatigue | 38 (16.5) | 6 (1.5) | **<0.001** |
| Neurological symptoms | 33 (14.4) | 10 (2.5) | **<0.001** |
| Respiratory symptoms | 20 (8.7) | 18 (4.5) | **0.038** |
| Psychological symptoms | 20 (8.7) | 11 (2.8) | **0.002** |
| Muscle and joint pains | 22 (9.6) | 9 (2.3) | **<0.001** |
| Loss of taste – smell | 20 (8.7) | 1 (0.25) | **<0.001** |
| Gastrointestinal symptoms | 12 (5.2) | 7 (1.8) | **0.026** |
| Dermatological disorders | 9 (3.9) | 3 (0.75) | **0.011** |
| Palpitations and cardiac disorders | 4 (1.7) | 1 (0.25) | 0.062 |
| Other symptoms | 3 (1.3) | 1 (0.25) | 0.141 |

*P values were computed by the Fisher’s exact test.

**Symptoms’ multivariable analysis**

In multivariable analysis the occurrence of symptoms during previous SARS-CoV-2 infection emerged as the most important predictor of subsequent Long COVID-19 symptoms in the Primary Care Setting (**Table 5**). The risk of Long COVID-19 also significantly increased with age, while gender and pre-existing diseases had no effect, even when asthma, allergies, or asthmatic bronchitis (**Table 6**).

Results of multivariable analysis were substantially confirmed, when separately considering the main symptoms. As an exception, while the risk of psychological and neurological symptoms, and abnormal fatigue significantly increased with age, an opposite trend was observed for respiratory diseases. Of note, the impact of symptoms during the previous infection had a very large impact on abnormal fatigue (OR=12.22, 95% CI=5.01-29.78, p=<0.001) and neurological symptoms (OR=6.61, 95% CI=3.13-13.94, p=<0.001), while the effect on respiratory (OR=2.11, 95% CI=1.07-4.13, p=0.030) and psychological (OR=3.08, 95% CI=1.42-6.67, p=0.004), although significant, was less pronounced. Again, gender and pre-existing diseases were devoid of significant effects.

**Table 5**. **Independent effect of gender, age, pre-existing diseases, and symptoms during SARS-CoV-2 infection on Long COVID-19 symptoms in the primary care setting.**

| **Response variable** | **Explanatory variables** | **OR (95% CI)** | ***p*-value** |
| --- | --- | --- | --- |
|  | Gender (Females vs Males) | 1.18 (0.79-1.76) | 0.421 |
|  |  |  |  |
|  | Age (years) |  |  |
| **At least one Long COVID-19** | 0-5 | 1 (reference) |  |
| **symptom** | 6-10 | 1.41 (0.84-2.37) | 0.194 |
|  | 11-16 | **2.18 (1.31-3.62)** | **0.003** |
| (153) |  |  |  |
|  | Pre-existing diseases (yes vs no) | 1.11 (0.58-2.12) | 0.746 |
|  |  |  |  |
|  | Symptomatic acute infection (yes vs no) | **6.57 (4.36-9.9)** | **<0.001** |
|  | Gender (Females vs Males) | 1.31 (0.67-2.55) | 0.424 |
|  |  |  |  |
| **Abnormal fatigue** | Age |  |  |
| (44) | 1-5 years | 1 (reference) |  |
|  | 6-10 | 2.85 (0.87-9.34) | 0.083 |
|  | 11-16 | **7.05 (2.35-21.12)** | **<0.001** |
|  |  |  |  |
|  | Pre-existing diseases (yes vs no) | 1.52 (0.62-3.74) | 0.355 |
|  |  |  |  |
|  | Symptomatic acute infection (yes vs no) | **12.22 (5.01-29.78)** | **<0.001** |
|  | Gender (Females vs Males) | 1.67 (0.85-3.24) | 0.132 |
|  |  |  |  |
|  | Age (years) |  |  |
| **Neurological symptoms** | 1-5 | 1 (reference) |  |
| (43) | 6-10 | **5.27 (1.47-18.94)** | **0.011** |
|  | 11-16 | **8.73 (2.54-29.98)** | **0.001** |
|  |  |  |  |
|  | Pre-existing diseases (yes vs no) | 0.79 (0.28-2.18) | 0.646 |
|  |  |  |  |
|  | Symptomatic acute infection (yes vs no) | **6.61 (3.13-13.94)** | **<0.001** |
|  | Gender (Females vs Males) | 0.98 (0.5-1.91) | 0.954 |
|  |  |  |  |
|  | Age (years) |  |  |
| **Respiratory symptoms** | 0-5 | 1 (reference) |  |
| (38) | 6-10 | **0.32 (0.14-0.72)** | **0.006** |
|  | 11-16 | **0.23 (0.09-0.58)** | **0.002** |
|  |  |  |  |
|  | Pre-existing diseases (yes vs no) | 1.14 (0.32-4.03) | 0.840 |
|  |  |  |  |
|  | Symptomatic acute infection (yes vs no) | **2.11 (1.07-4.13)** | **0.030** |
|  | Gender (Females vs Males) | 1.61 (0.75-3.43) | 0.217 |
|  |  |  |  |
|  | Age (years) |  |  |
| **Psychological symptoms** | 1-5 | 1 (reference) |  |
| (31) | 6-10 | 1.28 (0.39-4.16) | 0.682 |
|  | 11-16 | **3.79 (1.36-10.52)** | **0.011** |
|  |  |  |  |
|  | Pre-existing diseases (yes vs no) | 0.78 (0.22-2.74) | 0.699 |
|  |  |  |  |
|  | Symptomatic acute infection (yes vs no) | **3.08 (1.42-6.67)** | **0.004** |

**Table 6**. **Independent effect of gender, age, allergic diseases, and symptoms during SARS-CoV-2 infection on respiratory symptoms in the primary care setting.**

| Response variable | Explanatory variables | **OR (95% CI)** | ***p*-value** |
| --- | --- | --- | --- |
|  | Gender (Females vs Males) | 2.48 (0.06-108.85) | 0.638 |
|  |  |  |  |
|  | Age (years) |  |  |
| **Respiratory symptoms (n=38)** | 0-5 | 1 (reference) |  |
|  | 6-10 | 0.07 (0-2.84) | 0.162 |
|  | 11-16 | / |  |
|  |  |  |  |
|  | Allergic diseases (yes/no) | 1.29 (0.04-39.72) | 0.883 |
|  |  |  |  |
|  | Symptomatic acute infection (yes vs no) | 2.15 (0.07-61.42) | 0.654 |

**DISCUSSION**

Just recently, as Long COVID-19 came to be recognized in adults [8], growing evidence arises that it could also occur in children [9]. Nowadays studies on Long COVID-19 in pediatric population are scanty and heterogeneous in design, inclusion criteria, outcomes, and follow-up time, thus being difficult to compare. Actually, a recent review by Zimmermann et al. shows that the prevalence of Long COVID-19 symptoms in children varied considerably between studies, from 4 to 66% [10]. At our knowledge, ours is the first study on Long COVID-19 involving more than 600 children diagnosed with COVID-19 without need of hospitalization. Majority of studies focus uniquely on hospitalized population, but in pediatric age we know SARS-CoV-2 infection runs often with mild or no symptoms, thus a great number of infected children are excluded from analyses, because they have a greater risk of having Long COVID-19 symptoms overlooked [11].

Acute COVID-19 infection prevalence in children assisted in primary care was of 6% in our study. Even though this sample cannot be held as representative of the whole Italian Pediatric Population, our data are comparable to the ones published by Epicentro (Istituto Superiore di Sanità) in the December 2021 Report, which shows a COVID-19 prevalence of 6.5% in the Italian Cases from 0 to 9 years [12].

The incidence of Long COVID-19 is substantial in the Italian pediatric population. In the present study cumulative incidence amounted to 24.3% (95 CI% 21.0-27.9%) among children recovered from SARS-CoV-2 infection without need of hospitalization. In our study we added a small hospitalized series for a comparison; however, it comes from an only one Italian Hospital, thus it is not able to represent an accurate epidemiological data.

The probability of developing Long COVID-19 increases with the severity of previous SARS-CoV-2 infection, as also been demonstrated in adults [13]: in our cohort, prevalence goes from 11.5% in asymptomatic children to 46.5% in children with symptomatic infection, to 58% in hospitalized children.

Being symptomatic during the SARS-CoV-2 infection increased six folds the risk of having at least one symptom of long COVID-19, particularly respiratory, neurological, and psychological symptoms. This requires the PCP to monitor all patients who have had COVID-19, especially the symptomatic ones during the acute phase, but also the asymptomatic ones. Knowing that putative explanations for these Long COVID-19 symptoms include viral protein persistence in epithelial reservoirs, persistence of low-level inflammation, mitochondrial dysfunction, and virus-induced dysautonomia, it seems plausible that symptomatic children develop more frequently Long COVID-19 syndrome, as they typically experience a more virulent infection [14].

Long COVID-19 is far more frequent in hospitalized patients, but curiously abnormal fatigue has major prevalence in children who did not need hospitalization. This data contrasts with other studies reporting fatigue as the major symptom of Long COVID-19 after discharge [15-16]. This data could be due to the accuracy of the detection of symptoms made directly by the pediatrician in the interview with the parents or during the clinical visit.

Generally, children recruited in primary care developed less severe Long COVID-19 symptoms (fatigue, upper respiratory system symptoms), while hospitalized children showed high prevalence of cardiac involvement (~22%) and above all psychological symptoms (~37%): anxiety, fear in social relationships, depression, insomnia, and concentration difficulties.

According to the data available in literature, children aged 11-16 years have a greater risk of developing Long-COVID-19 symptoms, such as fatigue, anosmia, ageusia than the younger ones, aged 0-5 years [15,17]. However, this data could be unreliable as a consequence of the difficulty in the assessment of these symptoms in younger children. The frequency of respiratory disorders related to long COVID-19, instead, decreases with age. Younger children should be carefully monitored, because they could be more susceptible of severe respiratory complications.

In our study, gender and pre-existing diseases do not affect the occurrence of long COVID-19; also children without comorbidities can have a severe course for long COVD-19 symptoms.

The limitation of our study is that it relies on forms retrospectively filled by the PCP according to parents’ reported symptoms. Anyway, we believe that the information is reliable because PCPs in Italy have a very close relationship with the children’s families and the data have been carefully collected.

**CONCLUSION**

For many months after the breakout of the pandemic, parents’ worries about their children’s symptoms were minimized, often labelled as “psychological issues” [16]. Our study demonstrates that Long COVID-19 is a reality in pediatric age and could involve even patients with mild or no acute symptoms. The prevalence of Long COVID-19 is higher in symptomatic children, with no difference between males and females. Symptoms of Long COVID-19 are less severe in children assisted in primary care than in children hospitalized during acute illness. Our study provides useful information that stress the importance of monitoring primary care patients after acute COVID-19 infection, in particular younger children for respiratory symptoms and older children for psychological symptoms or fatigue.

Moreover, our results stress the importance of vaccination programs in pediatric population, also in order to avoid the consequences of Long COVID-19 syndrome.

**ABBREVIATIONS**

**SARS-CoV-2:** Severe Acute Respiratory Syndrome CoronaVirus 2

**COVID-19:** CoronaVirus Disease 2019

**PCP:** Primary Care Pediatrician

**DECLARATIONS**

**Ethics approval and consent to participate**

The present study was approved by the Regional Ethics Committee of Liguria on the 16^th^ of June 2021 with protocol number 419/2021 - id 11611. Written informed consent was obtained from all parents and/or legal guardian of enrolled children. All the methods were carried out in accordance with the ethical standards as laid down in the 1964 Declaration of Helsinki and its later amendments.

**Consent for publication**

Informed consent was obtained from all patients’ parents

**Availability of Data and Materials**

The datasets used and/or analyzed during the current study are available from the corresponding author on reasonable request.

**Competing interests**

The authors declare that they have no competing interests.

**Funding**

The study has been pursued with no sources of funding to declare.

**Authors’ contributions**

EB, VF, GT and GV have made substantial contributions to conception and design of the study, interpretation of data, and revising the manuscript critically for important intellectual content; GM and RV, have made substantial contributions to conception and design of the study, acquisition of data and analysis, interpretation of data, and drafting the manuscript; AS and AD have made substantial contributions to acquisition of data and analysis, and drafting the manuscript.

All authors have given the final approval of the version to be published.

**Acknowledgments**

We sincerely thank the Primary Care Pediatrician Research Group for the pivotal contribution to our study.

**REFERENCES**

1. Onder G, Floridia M, Giuliano M, Lo Noce C, Tiple D, Bertinato L, Mariniello R, Laganà MG, Della Vecchia A, Gianferro R, De Feo A, Cosenza P, Di Corcia T, Gianfranco Gensini G, Palummeri E, Frabetti C, Aliberti S, Campana A, Carfì A, Landi F, Rossi A, Cherubini A, Uccelli A, Barisione E, Benedetti L, Bartoloni A, Bonfanti P, Carlesimo M, Guaraldi G, Milic J, Leonardi S, Petrosillo N, Tarsia P. Indicazioni ad interim sui principi di gestione del Long-COVID. Versione del 1° luglio 2021. Roma: Istituto Superiore di Sanità; 2021. (Rapporto ISS COVID-19n. 15/2021). http://www.iss.it . Accessed 30 October 2021
2. Carfì A, Bernabei R, Landi F; Gemelli Against COVID-19 Post-Acute Care Study Group. Persistent Symptoms in Patients After Acute COVID-19. JAMA. 2020;324(6):603-605. doi:10.1001/jama.2020.12603. DOI: 10.1001/jama.2020.12603.
3. Huang C, Huang L, Wang Y, Li X, Ren L, Gu X, Kang L, Guo L, Liu M, Zhou X, Luo J, Huang Z, Tu S, Zhao Y, Chen L, Xu D, Li Y, Li C, Peng L, Li Y, Xie W, Cui D, Shang L, Fan G, Xu J, Wang G, Wang Y, Zhong J, Wang C, Wang J, Zhang D, Cao B. 6-month consequences of COVID-19 in patients discharged from hospital: a cohort study. Lancet. 2021 Jan 16;397(10270):220-232. doi: 10.1016/S0140-6736(20)32656-8. Epub 2021 Jan 8. PMID: 33428867; PMCID: PMC7833295. DOI: 10.1016/S0140-6736(20)32656-8
4. Wan Y-M, Deng X, Tan E-K, Olfattory disfunction and COVID-19, The Lancet Psychiatry, 2020 Aug;7(8):663. DOI: 10.1016/S2215-0366(20)30253-4
5. Velayudhan L, Aarsland D, Ballard C, Psychiatric and Neuropsychiatric syndromes and COVID-19, The Lancet Pshychiatry, 2020 Aug;7(8):663-664. DOI: 10.1016/S2215-0366(20)30291-1
6. <https://emergency.cdc.gov/han/2020/han00432.asp>. Accessed 17 March 2022
7. Götzinger F, Santiago-Garcia B, Lanaspa M, COVID-19in children and adolescents in Europe: a multinational, multicentre cohort study, The Lancet Child Adolescent Health, 2020 Sep;4(9):653-661. DOI: 10.1016/S2352-4642(20)30177-2
8. Greenhalgh T, Knight M, A’Court C, Management of post-acute covid-19 in primary care, BMJ, 2020 Aug 11;370:m3026. DOI: 10.1136/bmj.m3026
9. Buonsenso D, Munblit D, De Rose C, Preliminary evidence on Long COVID in children, Acta Paediatr, 2021 Jul;110(7):2208-2211 DOI: 10.1111/apa.15870
10. Zimmermann P, Pittet LF, Curtis N, How Common is Long COVID in Children and Adolescents?, Pediatr Infect Dis J, 2021 Dec 1;40(12):e482-e487. DOI: 10.1097/INF.0000000000003328
11. Simpson F, Chew-Graham C, Lokugamage A, Long COVID in children: the perspectives of parents and children need to be heard, Br J Gen Pract, 2021 Apr 29;71(706):216. DOI: 10.3399/bjgp21X715769
12. <https://www.epicentro.iss.it/coronavirus/bollettino/Bollettino-sorveglianza-integrata-COVID-19_15-dicembre-2021.pdf>. Accessed 16 December 2021
13. Sudre CH, Murray B, Varsavsky T, Attributes and predictors of Long COVID, Nat Med, 2021 Apr;27(4):626-631. DOI: 10.1038/s41591-021-01292-y
14. Montani D, Savale L, Noel N, Meyrignac O, Colle R, Gasnier M, Corruble E, Beurnier A, Jutant EM, Pham T, Lecoq AL, Papon JF, Figueiredo S, Harrois A, Humbert M, Monnet X; COMEBAC Study Group. Post-acute COVID-19 syndrome. Eur Respir Rev. 2022 Mar 9;31(163):210185. doi: 10.1183/16000617.0185-2021. PMID: 35264409.
15. Osmanov IM, Spiridonova E, Bobkova P, Risk factors for Long covid in previously hospitalised children using the ISARIC Global follow-up protocol: A prospective cohort study, Eur Respir J, 2021 Jul 1;2101341. DOI: 10.1183/13993003.01341-2021
16. Brackel CLH, Lap CR, Buddingh EP, Pediatric Long-COVID: An overlooked phenomenon?, Pediatric Pulomonology, 2021 Aug;56(8):2495-2502. DOI: 10.1183/13993003.01341-2021
17. Asadi-Pooya AA, Nemati H, Shahisavandi M, Long COVID in children and adolescents, World J Pediatr, 2021 Oct;17(5):495-499. DOI: 10.1007/s12519-021-00457-6.
18. Buonsenso D, Fusco C, De Rose C, Long COVID in children: Partnerships between families and paediatricians are a priority for better care, J Paediatr Child Health, 2021 Jun 1;10.1111/jpc.15600. DOI: 10.1111/jpc.15600

**FIGURE LEGENDS**

**Figure 1a-1b.** Total number of patients = 629. Hospitalized patients are described in **Supplementary figure 1a-1b**.

**TABLE LEGENDS**

**Table 1.** No legend needed.

**Table 2.** Number of children recruited in primary care = 629. Number of children discharged from hospital = 60. Number of cases are reported with percentages in parentheses.

**Table 3.** Total number of patients = 629. Classes of ages are 0-5, 6-10, 11-16 years. Number of cases are reported with percentages in parentheses.

**Table 4.** Total number of patients = 629. Data are reported as number of cases with percent frequencies in parentheses.

**Table 5.** Total number of patients = 629. Odds ratios and *p*-values were computed by a multivariable logistic regression model. Results on the whole sample are described in **Supplementary Table 2**.

**Table 6.** Total number of the patients = 629. Allergic diseases are asthma, allergies, asthmatic bronchitis. Odds ratios and *p*-values were computed by a logistic regression model**.**

**SUPPLEMENTARY MATERIAL**

**Supplementary Table 1. Cumulative incidence of Long COVID-19 symptoms and signs, as a function of age, in hospitalized children.**

|  | **Age class** | | | |
| --- | --- | --- | --- | --- |
|  | **0-5 years**  **(n=32)** | **6-10 years**  **(n=9)** | **11-16 years**  **(n=19)** | ***p*-value*** |
| At least one Long COVID-19 symptom | 12 (38) | 7 (78) | 16 (84) | **0.001** |
|  |  |  |  |  |
| Psychological symptoms | 5 (15.6) | 5 (55.6) | 12 (63.2) | **0.001** |
| Palpitations and cardiac disorders | 3 (9.4) | 3 (33.3) | 8 (42.1) | **0.017** |
| Respiratory symptoms | 1 (3.1) | 4 (44.4) | 6 (31.6) | **0.002** |
| Neurological symptoms | 3 (9.4) | 1 (11.1) | 3 (15.8) | 0.857 |
| Gastrointestinal disorders | 1 (3.1) | 2 (22.2) | 3 (15.8) | 0.114 |
| Dermatological disorders | 3 (9.4) | 1 (11.1) | 2 (10.5) | 1.000 |
| Muscle and joint pain | 0 | 1 (11.1) | 2 (10.5) | 0.129 |
| Loss of taste/smell | 0 | 0 | 1 (5.3) | 0.467 |
| Other symptoms | 6 (18.7) | 4 (44.4) | 9 (47.4) | **0.080** |

**Supplementary Figure 1a-1b. Cumulative incidence of Long COVID-19 symptoms in the hospitalized patients, as a function of previous diseases.**

**Fig. 1a**

**
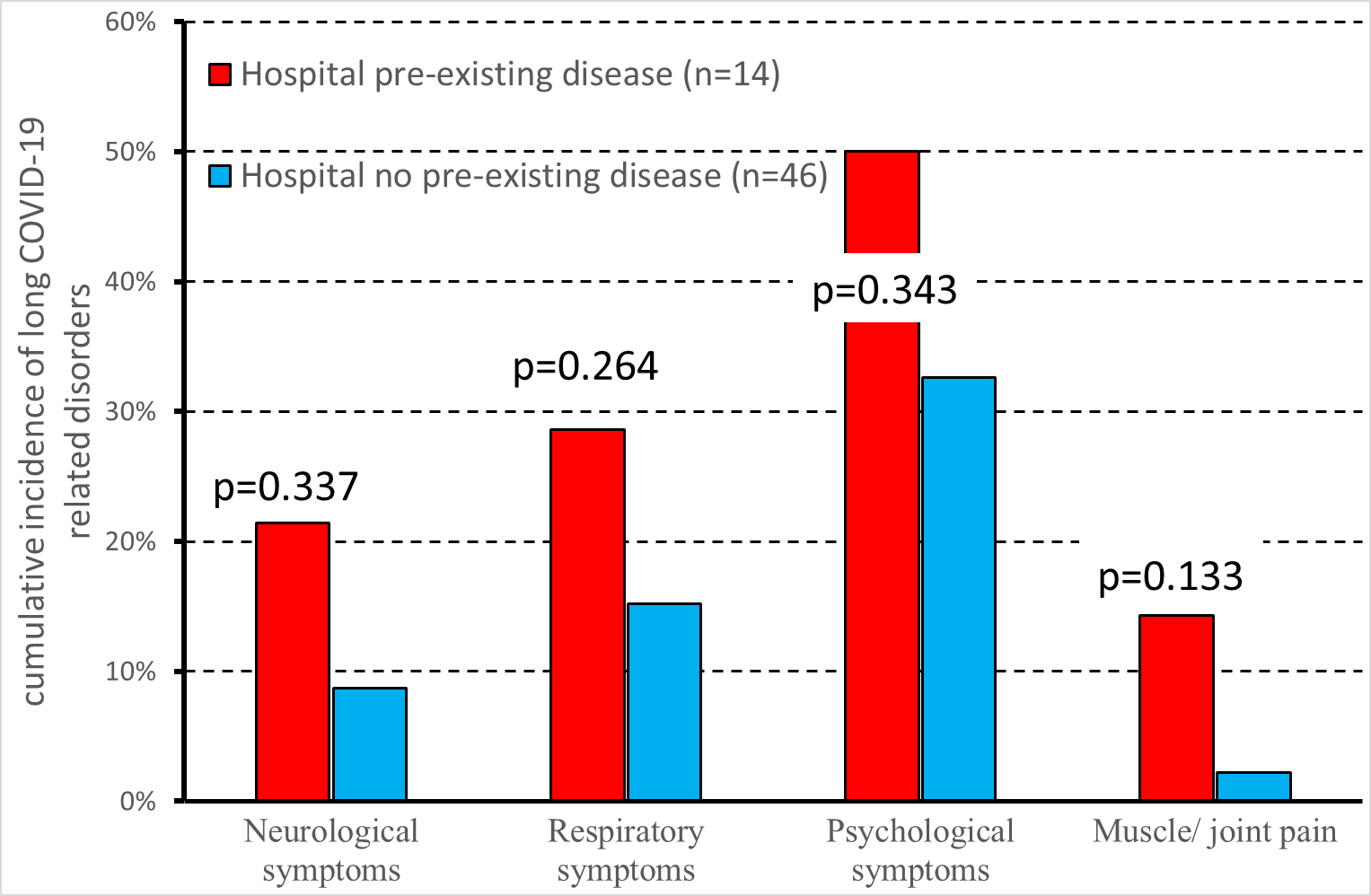
**

**Fig. 1b**

**
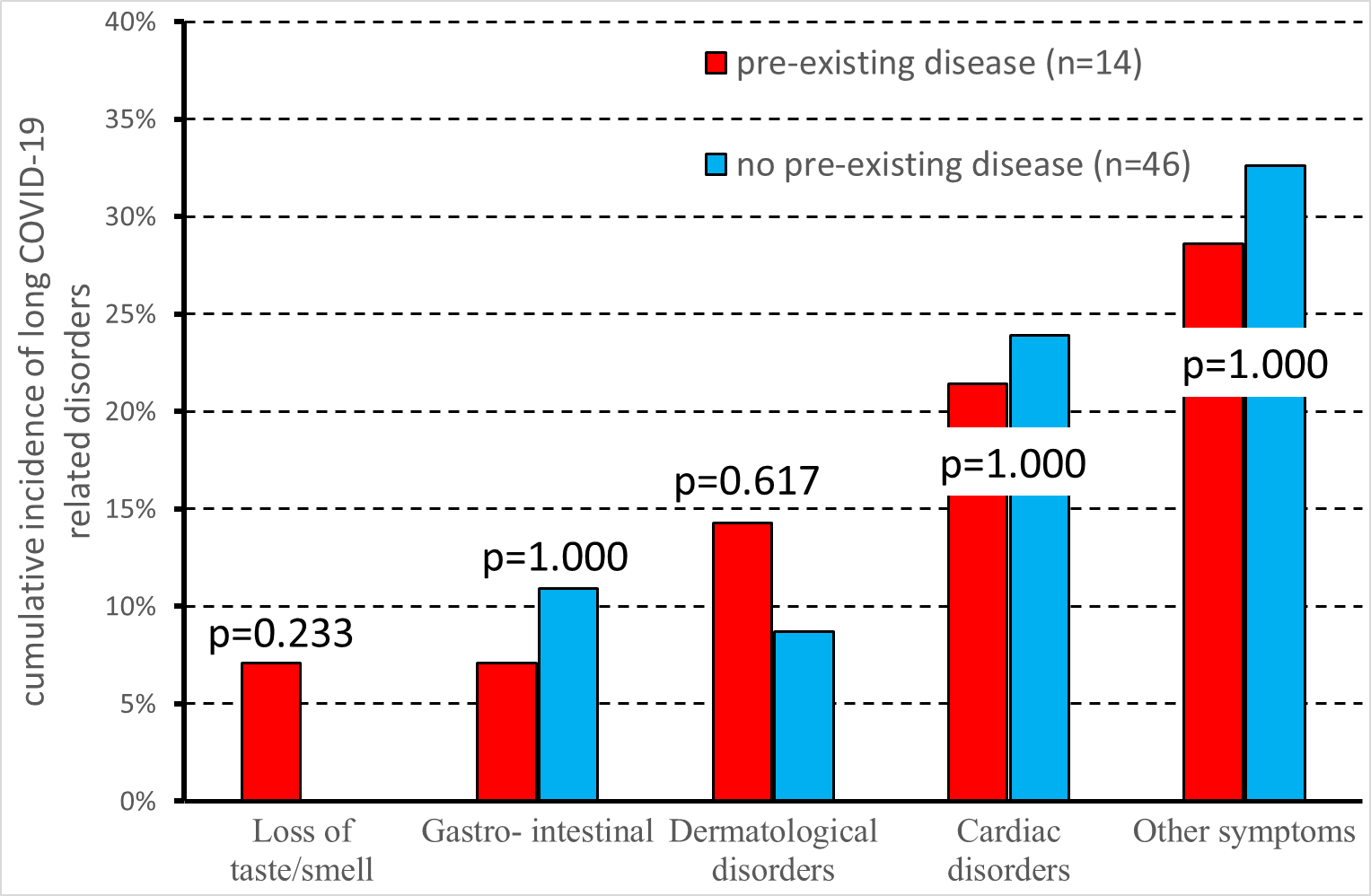
**

**Supplementary Table 2. Independent effect of setting, gender, age, and symptoms during SARS-CoV-2 infection on the Long COVID-19 symptoms.**

| **Response variable** | **Explanatory variables** | **OR (95% CI)** | ***p*-value** |
| --- | --- | --- | --- |
|  | **Setting (Hospital vs primary care)** | **2.49 (1.36-4.54)** | **0.003** |
|  |  |  |  |
|  | **Gender (Females vs Males)** | **1.89 (0.81-1.73)** | **0.369** |
|  |  |  |  |
|  | **Age (years)** | **1 (reference)** |  |
| **At least one long COVID-19** | **0-5** |  |  |
| **symptom** | **6-10** | **1.76 (1.08-2.88)** | **0.023** |
| ***(cases=188)*** | **11-16** | **2.86 (1.78-4.59)** | **<0.001** |
|  |  |  |  |
|  | **Symptoms during infection (yes vs no)** | **6.16 (4.14-9.18)** | **<0.001** |
|  | **Setting (Hospital vs primary care)** | **2.27 (1.02-5.03)** | **0.044** |
|  |  |  |  |
|  | **Gender (Females vs Males)** | **0.89 (0.49-1.61)** | **0.699** |
|  |  |  |  |
|  | **Age (years)** |  |  |
| **Respiratory disorders** | **0-5** | **1 (reference)** |  |
| ***(cases=49)*** | **6-10** | **0.60 (0.29-1.24)** | **0.169** |
|  | **11-16** | **0.54 (0.26-1.13)** | **0.102** |
|  |  |  |  |
|  | **Symptomatic acute infection (yes vs no)** | **1.96 (1.03-5.04)** | **0.044** |
|  | **Setting (Hospital vs primary care)** | **15.21 (6.98-33.14)** | **<0.001** |
|  |  |  |  |
|  | **Gender (Females vs Males)** | **1.61 (0.83-3.09)** | **0.155** |
|  |  |  |  |
|  | **Age (years)** |  |  |
| **Psychological disorders** | **1-5** | **1 (reference)** |  |
| ***(cases=52)*** | **6-10** | **1.66 (0.62-4.4)** | **0.311** |
|  | **11-16** | **4.37 (1.83-10.38)** | **0.001** |
|  |  |  |  |
|  | **Symptomatic acute infection (yes vs no)** | **2.55 (1.28-5.08)** | **0.008** |
|  | **Setting (Hospital vs primary care)** | **1.56 (0.62-3.93)** | **0.347** |
|  |  |  |  |
|  | **Gender (Females vs Males)** | **2.06 (1.1-3.86)** | **0.024** |
|  |  |  |  |
|  | **Age (years)** |  |  |
| **Neurological symptoms** | **1-5** | **1 (reference)** |  |
| ***(cases=50)*** | **6-10** | **2.91 (1.09-7.8)** | **0.033** |
|  | **11-16** | **4.74 (1.88-11.97)** | **0.001** |
|  |  |  |  |
|  | **Symptomatic acute infection (yes vs no)** | **6.91 (3.32-14.43)** | **<0.001** |

**SUPPLEMENTARY FIGURE LEGENDS**

**Supplementary Figure 1a-1b.** Total number of patients = 60.

**SUPPLEMENTARY TABLE LEGENDS**

**Supplementary Table 1.** Total number of patients = 60. Classes of ages are 0-5, 6-10, 11-17 years. Number of cases are reported with percentages in parentheses.

**Supplementary Table 2.** Analyses performed on the whole sample (n=689). Odds ratios and *p*-values were computed by a multivariable logistic regression model.

**APPENDIX**

**Questionnaire**

Questions indicated with * had a mandatory answer.

| **QUESTIONS FOR PRIMARY CARE PEDIATRICIAN** |
| --- |
| Total number of patients assisted* |
| How many patients underwent nasopharyngeal swab for COVID-19 diagnosis since the breakout of the pandemic until today? |
| How many patients were positive to COVID-19? |
| **QUESTIONS FOR PATIENT’S PARENTS** |
| Age of the patient**:***  1yrs 2 yrs 3 yrs 4 yrs 5 yrs 6 yrs 7 yrs 8 yrs 9 yrs 10 yrs 11 yrs 12 yrs 13 yrs 14 yrs 15 yrs 16 yrs |
| Sex*: Female Male |
| The child has tested negative from COVID-19* |
| For a month |
| For two or three month |
| For four or five month |
| For more than six month |
|  |
| Does the child suffer from chronic pathologies*?  YES NO  If YES, specify………………… |
| After COVID-19 acute infection, Has the child suffered more freqently from respiratory disorders*?  YES NO |
| If YES, specifiy among the reported***:** |
| Tonsillitis YES NO  Othitis YES NO  Pharingitis and/or Tracheitis YES NO  Bronchitis or Pulmonary Disorders YES NO |
| After COVID-19 acute infection, Has the child suffered more freqently from gastrointestinal disorders*?  YES NO |
| If YES, specifiy among the reported***:** |
| Diarrhea YES NO  Vomit YES NO  Abdominal colics YES NO |
| After COVID-19 acute infection, Has the child reported other symptoms*? |
| If YES, specifiy among the reported***:** |
| Anxiety and fear of social relationships YES NO |
| Depression YES NO |
| Learning disabilities YES NO |
| Eating disorders YES NO |
| Cephalalgy YES NO |
| Insomnia**.** YES NO |
| Palpitations YES NO |
| Joint and muscolar pain YES NO |
| Abnormal fatigue YES NO |
| Cutaneous disorders YES NO |
| Loss of hair YES NO |
| Loss of taste YES NO |
| Loss of smell**.** YES NO |
| Other symptoms YES NO  If YES specify **………………………………………………………..** |
